# Is COPD a Progressive Disease? A Long Term Bode Cohort Observation

**DOI:** 10.1371/journal.pone.0151856

**Published:** 2016-04-21

**Authors:** Juan P. de-Torres, Jose M. Marín, Víctor Pinto-Plata, Miguel Divo, Pablo Sanchez-Salcedo, Jorge Zagaceta, Javier J. Zulueta, Juan Berto, Carlos Cabrera, Bartolome R. Celli, Ciro Casanova

**Affiliations:** 1 Pulmonary Department, Clínica Universidad de Navarra, Pamplona, Spain; 2 Pulmonary Department, Hospital Universitario Miguel Servet, Instituto Aragones Ciencias Salud & CIBERES, Zaragoza, Spain; 3 Pulmonary Department, Brigham and Women’s Hospital. Harvard Medical School, Boston, MA, United States of America; 4 Pulmonary Department, Hospital Universitario Jose Negrín, Las Palmas de Gran Canaria, Spain; 5 Pulmonary Department, Hospital Ntra Sra de Candelaria, Tenerife, Spain; University of Athens Medical School, SWITZERLAND

## Abstract

**Background:**

The Global Initiative for Obstructive Lung Diseases (GOLD) defines COPD as a disease that is usually progressive. GOLD also provides a spirometric classification of airflow limitation. However, little is known about the long-term changes of patients in different GOLD grades.

**Objective:**

Explore the proportion and characteristics of COPD patients that change their spirometric GOLD grade over long-term follow-up.

**Methods:**

Patients alive for at least 8 years since recruitment and those who died with at least 4 years of repeated spirometric measurements were selected from the BODE cohort database. We purposely included the group of non survivors to avoid a “survival selection” bias. The proportion of patients that had a change (improvement or worsening) in their spirometric GOLD grading was calculated and their characteristics compared with those that remained in the same grade.

**Results:**

A total of 318 patients were included in the survivor and 217 in the non-survivor groups. Nine percent of survivors and 11% of non survivors had an improvement of at least one GOLD grade. Seventy one percent of survivors and non-survivors remained in the same GOLD grade. Those that improved had a greater degree of airway obstruction at baseline.

**Conclusions:**

In this selected population of COPD patients, a high proportion of patients remained in the same spirometric GOLD grade or improved in a long-term follow-up. These findings suggest that once diagnosed, COPD is usually a non-progressive disease.

## Introduction

The Global Initiative for Obstructive Lung Diseases (GOLD) defines chronic obstructive pulmonary disease (COPD) as a disease "characterized by persistent airflow limitation (AL) that is usually progressive" [1]. This implies that the forced expiratory volume in the first second (FEV_1_) in most patients with COPD decreases progressively over time. The GOLD document also grades the severity of AL and it has been assumed that most patients start in a less severe GOLD spirometric grade of AL and progress to more severe grades as the disease worsens.

Several recent studies challenge this concept reporting that lung function progression in COPD patients is heterogeneous, with only a minority of patients (between 20–30%) belonging to the “rapid decliners” group [2,3,4]. These studies were based on individual longitudinal data measured over a relatively short period of time (3–5 years) and have used the mean decline of groups using absolute FEV_1_ in ml as the yardstick. Few studies have related the change of lung function over time to disease progression. One report studied the change in GOLD spirometric grades in a COPD population, but the changes were evaluated for only one year, too short a time to express the progression of disease [5]. Agusti et al. [6] also explored longitudinal GOLD grade changes in the ECLIPSE cohort during 3 years, but only for the new ABCD grading and not for the GOLD spirometric grades of AL.

Since most clinicians use the GOLD spirometric grading of airflow obstruction to determine disease severity and progression, the present study was planned to explore the long-term behavior of GOLD spirometric grades of AL in a large sample of well-characterized COPD patients followed in clinics. The patients were classified into two groups: survivors (with at least 8 years of follow-up) and, to avoid the “survivor selection” bias, non-survivors (with at least 4 years of measurements before death).

## Patients and Methods

COPD patients participating in this study were part of the BODE international cohort, prospectively recruited and followed between January 1997 and December 2013 [7] in 1 hospital in the United States (Bay Pines VAMHCS, Florida) and 3 in Spain (Hospital Miguel Servet, Zaragoza; Clinica Universidad de Navarra, Pamplona and Hospital Nuestra Sra de La Candelaria, Tenerife). Regardless of disease severity, all patients regularly attending the pulmonary clinics in those hospitals were equally invited to participate in the study. None of the patients died while waiting to be included in the study. The detailed methodology of the BODE international cohort recruitment and follow up protocol has been previously described [7]. Patients were evaluated after enrolment and were seen every year or until death. The patient and family were contacted if the patient failed to return for appointments. Death from any cause was recorded and confirmed using death certificates in Spain, and the social security registry in the USA.

COPD was defined by a history of smoking at least 10 pack-years and FEV_1_/FVC ratio less than 0.70 after 400ug of inhaled albuterol [8]. Patients were excluded if they had a history of asthma, bronchiectasis, tuberculosis or other confounding diseases like severe congestive heart failure (Stage III through IV NYHA), obliterative bronchiolitis or diffuse panbronchiolitis. All COPD participants were clinically stable receiving standard medical treatment according to the American Thoracic Society/European Respiratory Society (ATS/ERS) guidelines [9].

The human-research review board at each institution approved the study and all patients signed an informed consent. (Comité de ética e investigación clínica de Aragón (CEICA), No.: 96/0115).

### Clinical and physiological parameters measurements

Trained personnel obtained the following information at the time of recruitment and at each visit thereafter: age, sex, spirometric values, mMRC dyspnea scale, six minute walk distance (6MWD), and the body mass index (BMI), calculated as the weight in kilograms divided by height in meters^2^. Patients were asked about their smoking history (age at initiation and discontinuation, as well as intensity), and if they still smoked (current or former) [10]. From this information we calculated the total smoking exposure and expressed it as pack-years.

Spirometry was performed following ERS/ATS guidelines [8]. FEV_1_ and FVC were measured and post bronchodilator (after 400ug of inhaled albuterol) values were recorded. All BODE sites follow strict quality control protocols of spirometry data.

For the purpose of the present study we selected patients that were alive at least for 8 years with spirometric measurements at baseline and at year 8. We arbitrarily selected 8 years of follow up for the survivors' group in an attempt to include a significant number of patients that were followed for a long period of time (in this case 318pts). To avoid the “survivor selection” bias, we also included a group of non survivors to determine their spirometric course. This group included patients who had 3 or more spirometries done for 4 years before their death to determine whether they differed in behavior from those that survived. Patients in this group had to have the last spirometry completed within a year of the date of death.

A patient was considered to not have progressed if he/she remained in the same spirometric GOLD grade. On the other hand, for progression there had to be a change from a lesser to a more severe GOLD spirometric grade of AL whereas for improvement the patient had to have changed to a lesser grade of AL. The GOLD spirometric grades used were as follows: grade I: FEV1%>80%, II: FEV1% 50–79, III: 30–49 and IV FEV_1_%<30 [1].

### Statistical analysis

Quantitative data with a normal distribution were expressed using the mean and the standard deviation (SD). Quantitative data with non-normal distribution were described with the median and the interquartile range (IQR). Qualitative data were described using relative frequencies. Comparison between groups we performed using Pearson chi-square, Student *t* or Mann-Whitney U according to the variables type and distribution. Bonferroni correction was used when multiple comparisons were made.

Significant levels for all tests were established as a two-tailed p-value < 0.05. Calculations were made with SPSS version 20.0 Inc. (IBM, Armonk, New York, USA).

## Results

**Fig 1:**
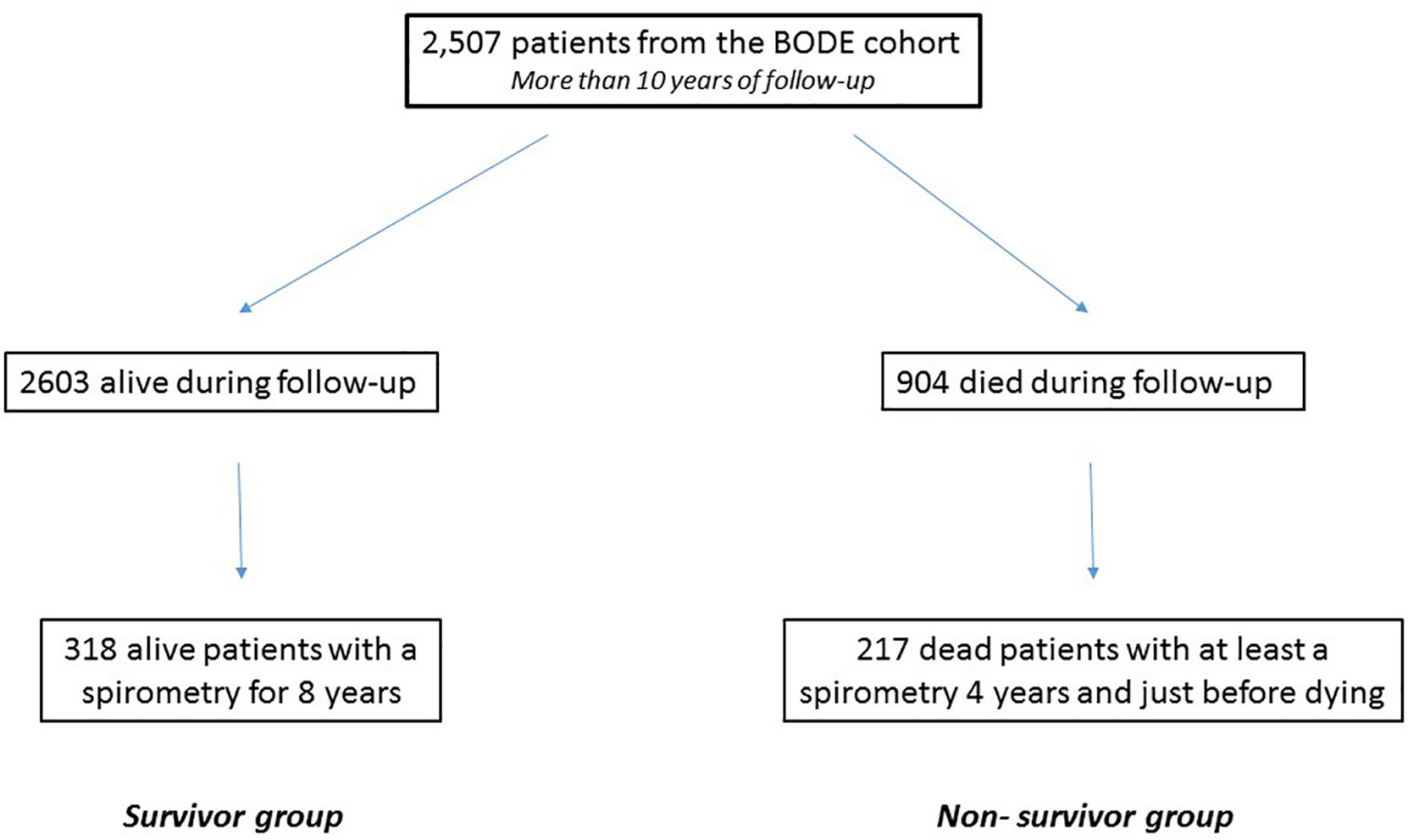
Flow chart of the patients participating in the study.

Fig 1 shows the flow chart of the patients included in the present study. A total of 318 COPD patients constituted the survivor group (followed for a minimum of 8 years) and 217 formed the non-survivor group (followed for a minimum of 4 years before they died). The clinical, physiological and pharmacological characteristics of the patients are shown in Table 1. The cohort consisted primarily of current and former smoking men in their 6^th^ decade. The spirometric staging included patients in all stages, but with a majority in GOLD spirometric grades II-III as is common in clinical pulmonary practice.

Eighty one percent of the patients in the survivor group and 79% in the non-survivor group remained in the same GOLD spirometric grade of AL during the observation period. Importantly, 9% of the survivors and 11% of the non-survivors actually improved their GOLD spirometric grade of AL. There was no difference in the proportion of patients that either improved or remained stable between survivors and non-survivors.

**Table 1:** Baseline characteristics of all COPD patients included in this study.

Clinical and Physiological Characteristics	*n = 535*
**Patients by GOLD stages I-II-III-IV (%)**	21,41,28,10
**Age in years (SD)**	68(9)
**Gender (male%/female%)**	(92/8)
**BMI in kg/m**^**2**^ **(SD)**	27(5)
**Pack-years (IQR)**	60(40–90)
**Smoking at the time of enrollment %**	30
**Quitting smoking during follow up (%)**	74
**FEV**_**1**_**% (SD)**	59(23)
**FVC% (SD)**	97(20)
**FEV1/FVC**	53(11)
**TREATMENT (%)**	
**NONE**	14
**LAMA**	19
**LABA**	2
**LABA+LAMA**	5
**ICS+LABA**	17
**LAMA+LABA+ICS**	43
**Theophylline**	0

BMI: Body Mass Index; FEV1: forced expiratory volume in the first second; FVC: forced vital capacity; LAMA: long acting muscarinisc agents; LABA: long acting beta 2 agonists agents; ICS: inhaled corticosteroids

**Fig 2:**
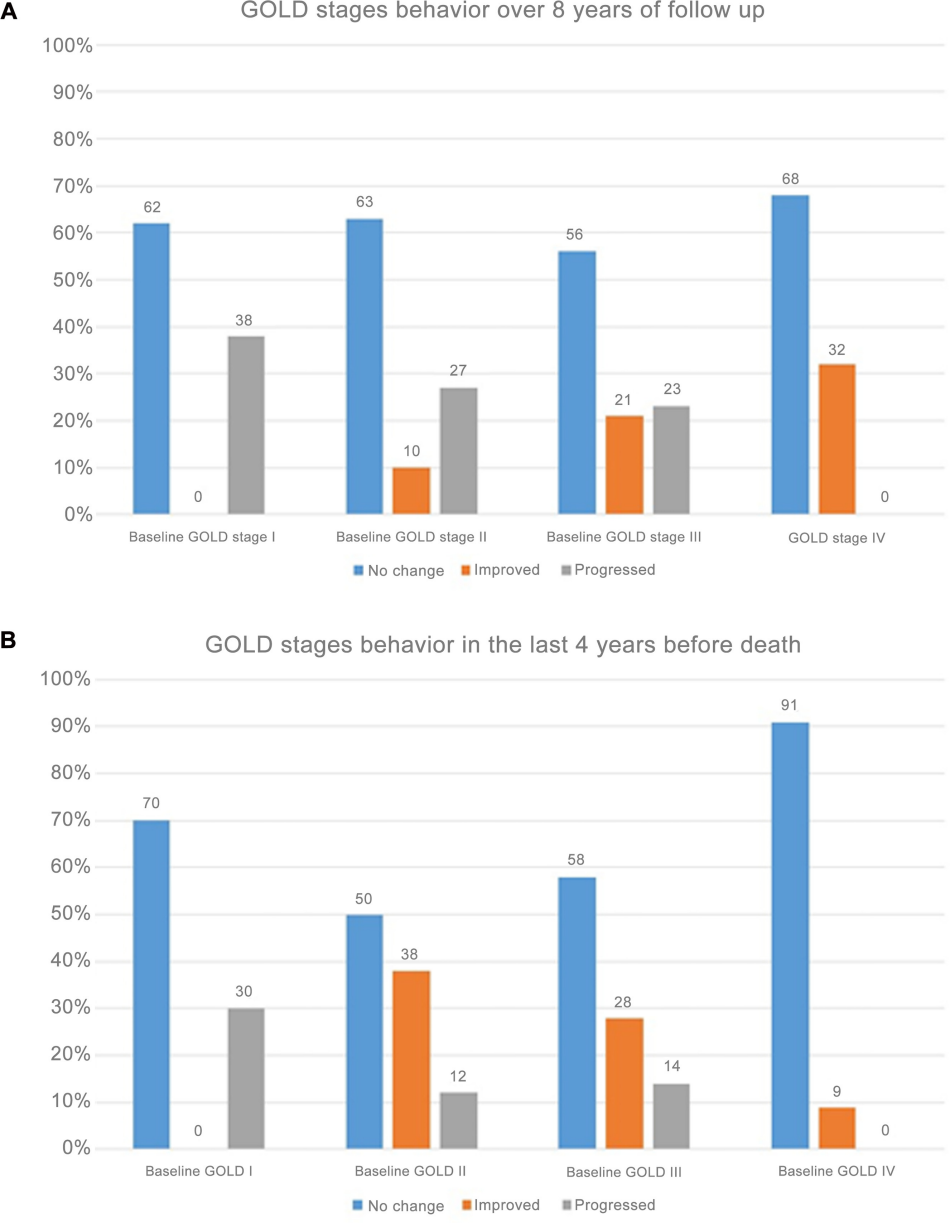
Behavior of baseline GOLD stages in each stage: **Panel A** for the survivors over at least 8 years of follow up and **Panel B** for the non survivors over at least 4 years of follow up before death.

Fig 2 shows the behaviour of the COPD patients in terms of progression, non-progression or improvement now classified by each spirometric GOLD grade. In the survivors group (Panel A), the proportion of patients that remained unchanged was similar across GOLD spirometric grades but surprisingly, a higher proportion of patients improved in the more severe grades of obstruction. In the non-survivor group (Panel B) a high percentage of patients remained unchanged or improved but most importantly, only a small percentage declined before death occurred.

The clinical, physiological and pharmacological characteristics of the patients from the survivor group that did not progress, progressed and improved are shown in Table 2. Statistical significant differences were found only in the baseline GOLD spirometric grades of AL, which was more severe in the group that improved.

**Table 2:** Baseline characteristics of the survivor COPD patients included in this study.

Clinical and Physiological Characteristics	*No progression*	*Progression*	*Improved*	*p* value	
	*n = 195*	*n = 92*	*n = 31*	*	**
**Patients by GOLD stages**					
**I-II-III-IV (%)**	30,50,17,3	38,46,16,0	0,48,42,10	0.42	<0.001
**Age in years (SD)**	67(10)	68(9)	67(9)	0.20	0.97
**Gender (male%)**	(95)	(90)	(93)	0.29	0.56
**BMI in kg/m**^**2**^ **(SD)**	27 (4)	27 (5)	28 (5)	0.68	0.87
**Pack-years (IQR)**	54(39–77)	60(42–84)	50(40–84)	0.27	0.53
**Smoking at the time of enrollment %**	34	37	29	0.51	0.68
**Quitting smoking during follow up (%)**	76	61	85	0.08	0.45
**FEV**_**1**_**% (SD)**	69(21)	67(20)	54(18)	0.81	<0.001
**FVC% (SD)**	102(19)	101(18)	92(25)	0.82	0.02
**FEV1/FVC**	56(10)	55(10)	51(12)	0.84	0.04
**TREATMENT (%)**					
				0.25	0.13
**NONE**	18	6	14		
**LAMA**	20	19	9		
**LABA**	2	0	5		
**LABA+LAMA**	5	2	9		
**ICS+LABA**	16	19	14		
**LAMA+LABA+ICS**	39	54	45		
**Theophylline**	0	0	4		

GOLD: Global Initiative for Obstructive Lung Disease; BMI: Body Mass Index; FEV1: forced expiratory volume in the first second; FVC: forced vital capacity; LAMA: long acting muscarinisc agents; LABA: long acting beta 2 agonists agents; ICS: inhaled corticosteroids

* Progression vs. No progression.

**Improved vs. No progression

Table 3 shows the clinical, physiological and pharmacological characteristics of the patients from the non-survivor group that did not progress, progressed and improved. No significant statistical differences were found between groups.

**Table 3:** Baseline characteristics of the non-survivor COPD patients included in this study.

Clinical and Physiological Characteristics	*No progression*	*Progression*	*Improved*	*p* value	
	*n = 129*	*n = 65*	*n = 23*	*	**
**Patients by GOLD stages**					
**I-II-III-IV (%)**	5,24,41,30	22,38,40,0	0,32,52,16	0.67	0.46
**Age in years (SD)**	67(8)	71(8)	71(7)	0.03	0.04
**Gender (male%)**	(93)	(92)	(92)	0.57	0.57
**BMI in kg/m**^**2**^ **(SD)**	26 (5)	26 (5)	26 (4)	0.68	0.87
**Pack-years (IQR)**	65(43–100)	70(50–96)	55(35–90)	0.27	0.53
**Current Smoking %**	25	30	13	0.23	0.15
**Quitting smoking during follow up (%)**	78	70	87	0.39	0.24
**FEV**_**1**_**% SD)**	43(19)	55(20)	49(17)	0.59	0.81
**FVC%(SD)**	84(21)	94(17)	87(23)	0.82	0.56
**FEV**_**1**_**/FVC**	47(11)	52(11)	48(13)	0.48	0.84

GOLD: Global Initiative for Obstructive Lung Disease; BMI: Body Mass Index; FEV1: forced expiratory volume in the first second; FVC: forced vital capacity;

* Progression vs. No progression.

**Improved vs. No progression

Table 4 shows the cause of death by baseline GOLD spirometric grades. Patients in GOLD spirometric grades I and II died primarily from cardiovascular disease and cancer, and those in grades III and IV died from respiratory failure.

**Table 4:** Cause of death in each GOLD baseline stage category represented by % of patients.

Cause of death	*GOLD I*	*GOLD II*	*GOLD III*	*GOLD IV*
**Cardiovascular disease %)**	23	11	7	2
**Lung Cancer %)**	31	25	10	9
**Other cancer %)**	31	13	12	5
**End stage COPD %)**	8	22	55	53
**Unknown %)**	8	26	11	19
**Infectious(pneumonia/sepsis)(%)**	0	2	5	12

## Discussion

There are two novel findings in this long term observational study of a large sample of COPD patients from the BODE cohort. First, that COPD is usually a non-progressive disease with less than 30% of the patients having a worsening of their GOLD spirometric grades of AL over at least 4 years of follow-up. Second, approximately 10% of the patients improved their spirometric values sufficiently to have an improvement in their GOLD spirometric grades of AL. These findings should contribute to challenge the current negative paradigm by providing a more positive perspective of a disease usually considered to be progressive.

Traditionally, and based primarily on the epidemiological observation from Fletcher and Peto [11], COPD has been viewed as a “usually progressive disease” [1] where patients are diagnosed at less severe spirometric grades and then progress over time to more severe grades. [1, 12, 13, 14]. However, this concept has been recently challenged in several studies. Sanchez-Salcedo *et al*. [15] demonstrated that the lung function decline profile is similar in young COPD patients and in those with older age, a finding that is not consistent with a progressive deterioration of lung function over time. More evidence suggesting that lung function progression in COPD is heterogeneous has been provided by three important studies [2, 3, 4]. Casanova *et al*. [2] followed 751 patients from the BODE database for a mean time of 5 years, and found that only 18% were “fast decliners” with a mean FEV_1_ decline of 86 ml/year. Similar results were reported by Nishimura *et al*. [3], who analyzed a smaller sample of 261 patients also followed for 5 years, reporting a similar proportion (25%) of “rapid decliners” who had a mean FEV_1_ decline of 61ml/year. Vestbo *et al*. [4], also reported the analysis of 2163 patients from the ECLIPSE study followed for 3 years, and observed that at least 38% of the patients had a yearly mean absolute FEV_1_ decline greater than 38ml/year. All of these studies concentrated primarily on the factors associated with a rapid decline in lung function, even though all of them noted the existence of a large group of patients who declined minimally and a small group that actually showed improvement in their lung function. These studies focused on the mean and individual yearly absolute FEV_1_ change in ml and did not relate the magnitude of the changes to shifts in GOLD spirometric AL grades, thereby limiting the clinical implications of the findings.

More recently, Casanova *et al*. explored the CHAIN COPD Spanish cohort [5] and reported the one year longitudinal changes of GOLD spirometric grades of AL in this population [5]. They noted that 72% of the patients remained in the same GOLD spirometric grade of AL and 13% actually improved, but the changes over that short period of time were not related to mortality or other outcomes. Agusti *et al*. in the ECLIPSE study also explored the longitudinal behavior of the GOLD spirometric grades of AL over 3 years but only analyzed the new ABCD GOLD classification [6] and did not explore the relationship between those changes and outcomes.

The present study expands on these observations exploring the long term changes in GOLD grades in a large sample of well characterized COPD patients. It has the strength of including patients that were followed for at least 8 years (many up to 10–12 years). The purposeful inclusion of a second group of COPD patients who died after a minimum of 4 years of follow up, was designed to minimize the potential bias of including only survivors that would have a better course. The fact that the proportion of patients that improved the GOLD spirometric grade of AL was similar in survivors (with a longer follow up time) and non-survivors, and that the proportion of patients that remained stationary (62% and 65%) was also similar in both groups, would suggest that the improvement or stability observed is not spurious and is likely real.

The meaning of an improvement in GOLD spirometric grade has not been validated although several studies have consistently shown that the grades themselves are very good predictors of mortality [5]. The actual reasons for this cannot be discerned in this study since it was not planned to explore any intervention other than optimal COPD care, but it does raise the possibility that the current standard of care is effective and provides strong support for a positive attitude towards patients with this disease, independent of the severity of the condition. In this regard, we observed no special characteristic that helped identify patients likely to improve over time, except for the fact that the baseline FEV_1_ was lower in those patients who had the most improvement. We have no explanation for this observation, except that the proportion of patients who quit smoking was higher, and the total amount of cigarettes smoked was lower, in patients who remained stable or improved compared with those that worsened, although it failed to reach statistical significance.

The present study has several limitations. Firstly, the findings presented are restricted to the type of patients included in this cohort: mainly male COPD patients attending pulmonary clinics. We do not know if women and/or patients attending primary care clinics will have the same behavior. Secondly, patients were managed differently over time as our capacity to treat them was influenced by the advent of newer therapies. This may have influenced the rate of FEV_1_ decline. However, this is true of all observational cohorts which by and large reflect real life medicine. On the other hand, the large number of patients and the international nature of the study decreases the chance that a systematic bias may have been applied to individual patients according to the GOLD spirometric grading. Our data (Table 2) shows that all groups had the same proportion of treatments regimens, suggesting that at least in this cohort treatment option had little effect on spirometric GOLD grading changes. We also acknowledge that we have no precise information on what happens in between the recorded visits and we only present a simple bivariate analysis, with all the potential limitations this could have. Thirdly, the BODE international cohort did not prospectively record the yearly exacerbation rate that could have had an impact on the progression of lung function, a weakness that may limit the strength of our findings. However, if we evaluate the results of the ECLIPSE study, where the exacerbation rate had little effect on FEV_1_ decline (only 2 ± 0.5ml/year for each exacerbation) the potential effect of this limitation is small [4]. Lastly, we should not forget the potential role of the “size effect” on the change. We used a change of at least 1% to consider either an improvement or a deterioration, knowing that such a small change in lung function probably does not imply a clinically important difference. Acknowledging this important limitation, if we would have used a higher cut off threshold to consider a change, then a much smaller percentage of patients would have improved or deteriorated, giving further support to the main message of the present work that COPD is “mainly a non-progressive disease”.

In summary, the present study suggests that COPD is not markedly progressive in patients cared for in pulmonary clinics of tertiary university care centers, since a large proportion of patients attending clinics do not have changes, or actually improve, in their GOLD spirometric grades of AL. This finding changes the long-term perspective of the disease from a nihilistic one that limits the potential for improvement to a more positive one that can be used to motivate patients to adopt healthier lifestyles and remain adherent to their treatment.

## Supporting Information

S1 Dataset Dataset of the present manuscript. (SAV)
